# Pathogenic and Virulence Factor Detection on Viable but Non-culturable Methicillin-Resistant *Staphylococcus aureus*

**DOI:** 10.3389/fmicb.2021.630053

**Published:** 2021-03-25

**Authors:** Hua Jiang, Kan Wang, Muxia Yan, Qian Ye, Xiaojing Lin, Ling Chen, Yanrui Ye, Li Zhang, Junyan Liu, Tengyi Huang

**Affiliations:** ^1^Department of Haematology, Guangzhou Women and Children’s Medical Center, Guangzhou Medical University, Guangzhou, China; ^2^Center for Translational Medicine, The Second Affiliated Hospital of Shantou University Medical College, Shantou, China; ^3^School of Food Science and Engineering, Guangdong Province Key Laboratory for Green Processing of Natural Products and Product Safety, South China University of Technology, Guangzhou, China; ^4^School of Biological Science and Engineering, South China University of Technology, Guangzhou, China; ^5^Department of Civil and Environmental Engineering, University of Maryland, College Park, College Park, MD, United States; ^6^Department of Laboratory Medicine, The Second Affiliated Hospital of Shantou University Medical College, Shantou, China

**Keywords:** VBNC, MRSA, PMA-CPA, virulence detection, cross-priming amplification

## Abstract

Food safety and foodborne infections and diseases have been a leading hotspot in public health, and methicillin-resistant *Staphylococcus aureus* (MRSA) has been recently documented to be an important foodborne pathogen, in addition to its recognition to be a leading clinical pathogen for some decades. Standard identification for MRSA has been commonly performed in both clinical settings and food routine detection; however, most of such so-called “standards,” “guidelines,” or “gold standards” are incapable of detecting viable but non-culturable (VBNC) cells. In this study, two major types of staphylococcal food poisoning (SFP), staphylococcal enterotoxins A (*sea*) and staphylococcal enterotoxins B (*seb*), as well as the panton-valentine leucocidin (*pvl*) genes, were selected to develop a cross-priming amplification (CPA) method. Limit of detection (LOD) of CPA for *sea*, *seb*, and *pvl* was 75, 107.5, and 85 ng/μl, indicating that the analytical sensitivity of CPA is significantly higher than that of conventional PCR. In addition, a rapid VBNC cells detection method, designated as PMA-CPA, was developed and further applied. PMA-CPA showed significant advantages when compared with PCR assays, in terms of rapidity, sensitivity, specificity, and accuracy. Compared with conventional VBNC confirmation methods, the PMA-CPA showed 100% accordance, which had demonstrated that the PMA-CPA assays were capable of detecting different toxins in MRSA in VBNC state. In conclusion, three CPA assays were developed on three important toxins for MRSA, and in combination with PMA, the PMA-CPA assay was capable of detecting virulent gene expression in MRSA in the VBNC state. Also, the above assays were further applied to real samples. As concluded, the PMA-CPA assay developed in this study was capable of detecting MRSA toxins in the VBNC state, representing first time the detection of toxins in the VBNC state.

## Introduction

In the past few decades, public health has been a major issue for society. From various points of view, food safety and foodborne infections and diseases have been a leading area of concern. Among food safety problems, foodborne infections or diseases caused by food pathogens have made up the majority of the cases (Xu et al., 2021).

*Staphylococcus aureus* is an important foodborne pathogen that is responsible for a large variety of foodborne infections and diseases (Weese et al., 2010; Wang et al., 2012; Miao et al., 2017a,b, 2019). Capable of producing enterotoxins, it is also commonly found in food poisoning cases. Therefore, staphylococcal food poisoning (SFP) is a major concern in public health (Zhao et al., 2010a,b,c, 2011; Crago et al., 2012; Deng et al., 2015a,b; Yu et al., 2016). In addition, methicillin-resistant *S. aureus* (MRSA) is one of most widely distributed human and animal pathogens that produce a variety of toxins and cause a range of serious illnesses of the skin, soft tissue, bone, and bloodstream (Tacconelli et al., 2009). MRSA was previously limited as a clinical microorganism. However, in recent years, studies have reported that MRSA is strongly and closely associated with food safety. For example, the carriage of this pathogen by industrial staff and the prevalence of livestock-associated MRSA (LA-MRSA) have been clearly pointed out and well established as a foodborne pathogen (Liu et al., 2016a,b,c, 2017a,2017b,2017c,2017d, 2018a,2018b,2018c,2018d, 2019; Xie et al., 2017a,b). Apart from that, MRSA has seen an increase in numerous countries and emerged as an important pathogen, accounting for up to 40% of all *S. aureus* isolates in nosocomial infection, and has caused significant morbidity (Graveland et al., 2011). This is due to the effect of β-lactam resistance in *S. aureus*, which is mediated by the production of penicillin-binding protein 2a PBP2a or PBP2′ encoded by the *mecA* gene (Moellering, 2012). The development of resistance to β-lactam antibiotics has been a cause of concern among the medical community.

Standard identification for MRSA has been commonly performed in both clinical settings and routine food detection. In 1982, viable but non-culturable (VBNC) was first proposed. A large proportion of studies have been done on foodborne microorganisms, as once having failed to allow detection of the bacterial cells, the food sample is likely to be mistakenly determined to be safe or qualified. In addition to foodborne pathogens, laboratory researchers in clinical settings commonly fail to detect or identify the pathogens responsible in apparently infected patients. Culturability-based methodology has been significantly challenged as the “gold standard” when it comes to microbiological identification. Therefore, rapid detection of MRSA is imperative for both MRSA-related foodborne outbreaks and clinical diagnosis. Current methodologies available for detection of MRSA include standard clinical testing (such as disk susceptibility tests or broth dilution) and molecular methods (such as polymerase chain reaction and real-time fluorescence PCR) (Salisbury et al., 1997; Felten et al., 2002; Corrente et al., 2007). The former usually takes 1–2 days to obtain results. At the same time, PCR is time-consuming and requires highly trained personnel. In addition, the sensitivity of PCR can be compromised by PCR inhibitors present in a biological sample (Monteiro et al., 1997). Therefore, these methods are not appropriate for rapid screening. Recently, isothermal amplification methodologies have received much attention due to the omission of thermocyclers, simple protocols, and fast analysis, and they have an analytical performance that competes PCR. A recent innovation of isothermal nucleic acid amplification methodology, cross-priming amplification (CPA), was developed to detect the target DNA with exponential amplification (Xu et al., 2008, 2009, 2010a,2010b, 2011a,2011b, 2017, 2019; Xu Z. et al., 2012). Compared with PCR, this novel amplification assay can amplify the target nucleotide sequence isothermally in a short time, which is cost-effective and time-saving (Xu G. et al., 2012). This technique does not rely on sophisticated instruments or trained technicians but can be conducted in a water bath or heating block. In PCR methods, the progress of the reaction is usually verified by electrophoresis. Different from PCR, the CPA assay is able to produce massive amplicons with the obvious turbidity that can observed by nucleic acid-specific fluorescent dye with or without UV illumination to improve detection efficiency (Wang et al., 2014; Xu et al., 2020a). So far, the CPA protocol has been successfully applied in the detection of virus and harmful microbes, such as *Escherichia coli* O157:H7, *Mycobacterium tuberculosis*, *Enterobacter sakazakii*, *Salmonella enterica*, and *Yersinia enterocolitica* (Fang et al., 2009; Yulong et al., 2010; Zhang et al., 2015; Wang et al., 2018; Xu et al., 2020a,b).

In this study, the VBNC state formation of MRSA has been studied in different real food samples. After confirmation of the VBNC formation, the expression of pathogenic, virulence factors was performed on three enterotoxins and *pvl*, based on the PMA-CPA methodologies.

## Materials and Methods

### Strains and Targets

A total of 36 strains were included in this study, as shown in Table 1, including five MRSA strains, 13 MSSA strains, and 18 non-Staphylococci strains. All strains used in this study had been preliminarily identified, and all MRSA strains were identified at the species level using standard procedures as follows: first observation by colony morphology, Gram staining, and catalase test, followed by identification using the Vitek 2 automated system and the API-Staph commercial kit. Further determination of methicillin resistance was performed by susceptibility testing on oxacillin-screening agar, verified by latex agglutination for PBP2a and *mecA* detection by PCR as described previously. The MRSA and MSSA strains used in this study were previously detected by the toxins by PCR and further Sanger sequencing, which were found to carry different toxins. Thus, such strains are included in different detection assays in this study. Three pathogenic targets have been selected for primer design (Table 2). Staphylococcal enterotoxins A (*sea*) and staphylococcal enterotoxins B (*seb*) are the two most commonly detected enterotoxins in Staphylococci strains and are responsible for various foodborne cases. Another key toxin for Staphylococci is panton-valentine leucocidin (*pvl*), which is a cytotoxin produced by Staphylococcus and causes leukocyte destruction and tissue necrosis. *Pvl* is an important toxin for MRSA, and it has been found in the recent decade that the community-associated MRSA (CA-MRSA), commonly carrying type IV or V SCCmec, is highly associated with these toxins. In addition, ST398 is well known to be a major type for liverstock-associated MRSA (LA-MRSA), which has been reported to be primarily within CA-MRSA instead of hospital-associated MRSA (HA-MRSA), commonly carrying types I, II, and III SCCmec. Based on the correlation between toxins and CA-MRSA, the carriage of *pvl* is high in food-contaminated MRSA strains, as they are commonly CA-MRSA. The above three targets have been selected to be included in the following studies.

**TABLE 1:** Reference strains used and the results of CPA assays.

**Reference strains**	**No. of strains**	**CPA/PCR assays**				
		***mecA***	***femA***	***sea***	***seb***	***pvl***
*Staphylococcus aureus* (MRSA) 0314030635	1	+	+	−	−	−
*Staphylococcus aureus* (MRSA) 971311004	1	+	+	−	−	−
*Staphylococcus aureus* (MRSA) 0313113664	1	+	+	−	−	−
*Staphylococcus aureus* (MRSA) 0314030668	1	+	+	−	−	−
*Staphylococcus aureus* (MRSA) 10071	1	+	+	−	−	−
*Staphylococcus aureus* (MSSA) 132115, 0315022822, 0613120003, 0314020129	4	−	+	+	+	−
*Staphylococcus aureus* (MSSA) 0315011480	1	−	+	−	+	−
*Staphylococcus aureus* (MSSA) 130149	1	−	+	−	−	−
*Staphylococcus aureus* (MSSA) 132113, 0314030635, 971310004, 132112	4	−	+	−	−	−
*Staphylococcus aureus* (MSSA) 0315040330	1	−	+	−	−	+
*Staphylococcus aureus* (MSSA) 0713100037	1	−	+	−	−	+
*Staphylococcus aureus* (MSSA) 0314020556	1	−	+	−	−	+
*Staphylococcus aureus* (MSSA) 0315011480	1	−	+	−	−	+
*Escherichia coli O157* ATCC43895	1	−	−	−	−	−
*Escherichia coli O157* E019, E020, E043, E044	4	−	−	−	−	−
*Salmonella* ATCC29629, ATCC19585, ATCC14028, ATCC13076,	4	−	−	−	−	−
*Listeria monocytogenes* ATCC19118, ATCC19116, ATCC19114, ATCC19115, ATCC15313, ATCC19113	6	−	−	−	−	−
*Vibrio parahaemolyticus* ATCC27969, ATCC17802	2	−	−	−	−	−
*Lactobacillus casei*	1	−	−	−	−	−

**TABLE 2:** Primer sequence of CPA.

**Target gene**	**Primers**	**Sequence (5′-3′)**
*femA*	4s	TCAAATCGCGGTCCAGTG
	5a	AACCAATCATTACCAGCA
	2a/1s	TACCTGTAATCTCGCCAT AACATCGTTGTCTATACCT
	2a	TACCTGTAATCTCGCCAT
	3a	GGTAAATATGGATCGATATG
*mecA*	4s	GCGATAATGGTGAAGTAG
	5a	GATCAATGTTACCGTAGTT
	2a/1s	TTACGATCCTGAATGTTT ATGACTGAACGTCCGATA
	2a	TTACGATCCTGAATGTTT
	3a	TCTTTAACGCCTAAACTA
*sea*	4s	GCTTGTATGTATGGTGGT
	5a	CTGTAAATAACGTCTTGC
	2a/1s	GAAGATCCAACTCCTGAA GGCTAGACGGTAAACAAA
	2a	GAAGATCCAACTCCTGAA
	3a	TTCGTTTTAACCGTTTCC
*seb*	4s	ATTACTGTTCGGGTATTTG
	5a	TTCATAAGGCGAGTTGTT
	2a/1s	AATAGTGACGAGTTAGGT CTTTTGACGTACAAACTA
	2a	AATAGTGACGAGTTAGGT
	3a	CTAATTCTTGAGCAGTCACT
pvl	4s	GTTGGGATGTTGAAGCAC
	5a	TGGATAACACTGGCATTT
	2a/1s	GTCCAGCATTTAAGTTGC GGACCATATGGCAGAGAT
	2a	GTCCAGCATTTAAGTTGC
	3a	CATTTCATTACCATAAG

### Formation of the VBNC State

Culturing, incubation, and inoculation of MRSA and non-MRSA strains were performed using routine procedures. All strains were stored in −80°C and were streaked on TSB plates overnight at 37°C. Colonies were picked and then inoculated into fresh TSB medium to obtain different strains in log phase, ranging from 2 to 6 h. For genomic DNA extraction, bacterial cells after overnight culturing were used, and after centrifugation, DNA extraction was performed using the Dongsheng BioTech DNA extraction kit, according to their manufacturers’ instructions. All extracted DNA samples were qualified and confirmed by both under 260/280 and electrophoresis. For VBNC formation, the procedure has been conducted as described previously. In brief, an initial concentration of bacterial cells at 10^8^ CFU/ml was used, and the cells are kept at 4 and −20°C, respectively. CFU counting was performed first to obtain the growth curve, and 10 representative time points were selected. Then strains were grown strictly as described above, and for the selected time points, VBNC was confirmed. In addition, VBNC formation was also performed in the real food samples, such as Cantonese cake, as described previously. In brief, the same concentration of bacterial culture was inoculated into the food sample and was kept at 4 and −20°C, respectively. Similar time points were selected as above, and VBNC cells were further confirmed. For VBNC confirmation, total cell numbers, culturable cell numbers, and viable cell numbers were determined as described previously. The LIVE/DEAD BacLight^TM^ kit was used before performed subjecting to flow cytometry. CFU was counting for culturable cell numbers. After VBNC cells were obtained, introduction of PMA was performed as described previously. Further, DNA extraction was processed, and CPA was also performed for detection.

### Development of CPA Assays

After primer design on three targets, primer was synthesized. CPA reaction was carried out as described previously. The reaction volume is first 25 μl, and further 1 μl mixed chromogenic agent (containing 0.13 mM calcein and 15.6 mM MnCl_2_⋅4H_2_O) was added. For the reaction program, 63°C for 1 h then 80°C for 2 min was used.

### Optimization of CPA Assays

To optimize the CPA assays, different reaction times ranging from 30 to 90 min were used. The reaction volume was used as above.

Limit of detection (LOD) was determined on the CPA assays, and 10-fold serial dilutions of total genomic DNA were used. All experiments were performed in triplicate.

The specificity study on the CPA assay consists of two parts. The first one is the specificity of primers. The CPA was performed with any one of the five primers (4s, 5a, 2a, 1s, 2a, and 3a) omitted to confirm that every single primer is strictly and specifically required for the reaction. Second, the specificity of the primers set was confirmed by including five MRSA strains with 31 non-MRSA strains.

### Evaluation of CPA Assays

To evaluate the CPA reaction, PCR was also performed using all primers with the following program: 95°C for 3 min, 30 cycles of amplification at 95°C for 30 s, 55°C 30 s, 72°C for 1 min, and final amplification at 72°C for 7 min. Nuclease-free water was used as a negative control. All PCR products had been subjected to Sanger sequencing to confirm the highly conserved region used for CPA primers design.

### Determination of Results

For all nucleic acid isothermal amplification assays, the determination of results shows significant advantages when compared with traditional methods like PCR. In this study, two ways were used as a basis of comparison for the determination of results. First, amplicons were detected by agarose gel electrophoresis, and the bands were observed under UV light. Second, color change was observed and determined by using a mixture of chromogenic agents (MgCl_2_ and calcein), and the color green was an indicator of positive yielding.

### Confirmation of VBNC Formation and Application of PMA-CPA

The VBNC formation in both pure culture and real samples was described as above. After the VBNC cells were obtained, DNA extraction was performed, and PMA was introduced. Then the CPA assays on three target toxins were applied to determine the toxin expression in MRSA within VBNC state.

## Results

### Establishment of CPA Assays

According to the results, the CPA assays for all three targets studied were able to yield expected bands, demonstrating that the CPA assays were successfully developed. According to the optimization of the CPA assays, the reaction temperature as 63°C was found to be the optimal temperature. The time duration of 60 min was found to be the optimal reaction time. Under such conditions, the reproducibility was found to be optimal.

### Sensitivity of CPA Assays

**FIGURE 1:**
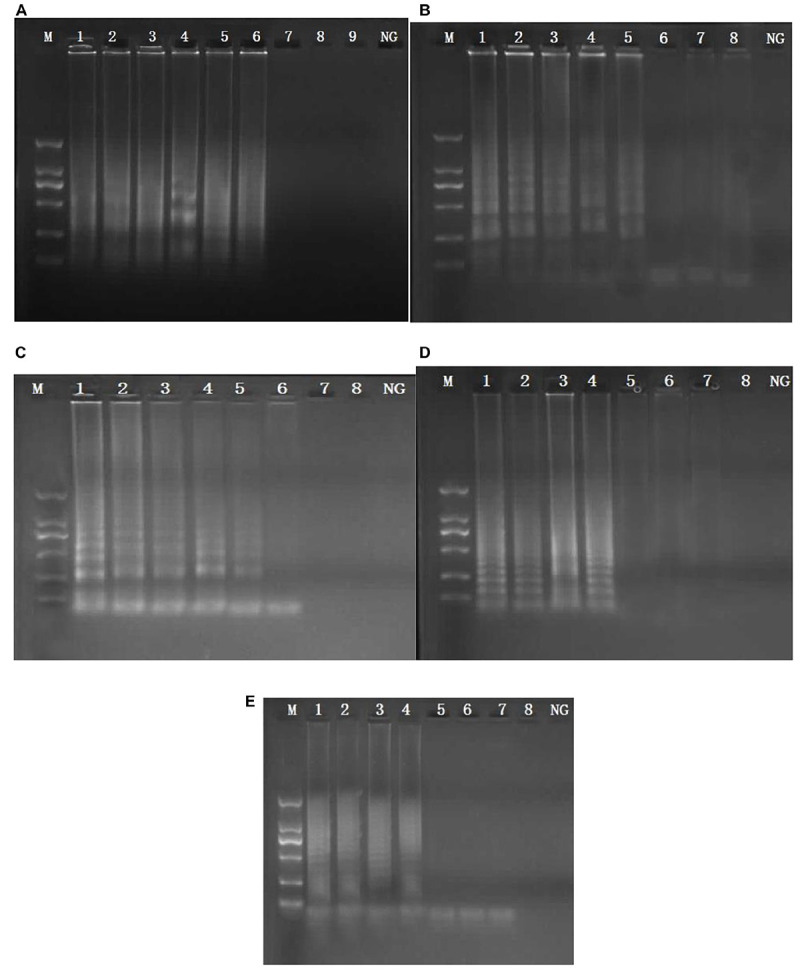
Sensitivity of the CPA assay in genomic DNA by 1.5% agarose gel electrophoresis. Sensitivity from 10071 of *femA* **(A)** and *mecA* **(B)** genes. M, DNA marker; lanes 1–8, 3.0 ng/μl, 300 pg/μl, 30 pg/μl, 3 pg/μl, 300 fg/μl, 30 fg/μl, 3 fg/μl, 300 ag/μl NG—negative control. Sensitivity from 0214010085 of *sea* **(C)**: M, DNA marker; lanes 1–8, 3.0 ng/μl, 300 pg/μl, 30 pg/μl, 3 pg/μl, 300 fg/μl, 30 fg/μl, 3 fg/μl, 300 ag/μl NG—negative control. Sensitivity from 0315011480 of *seb* **(D)**: M, DNA marker; lanes 1–8, 4.3 ng/μl, 430 pg/μl, 43 pg/μl, 4.3 pg/μl, 430 fg/μl, 43 fg/μl, 4.3 fg/μl, 430 ag/μl NG—negative control. Sensitivity from 0214010085 of *pvl* **(E)**: M, DNA marker; lanes 1–8, 3.4 ng/μl, 340 pg/μl, 34 pg/μl, 3.4 pg/μl, 340 fg/μl, 34 fg/μl, 3.4 fg/μl, 340 ag/μl NG—negative control.

The analytical sensitivity of the CPA assay for MRSA was measured using 10-fold serial dilutions of MRSA genomic DNA. The LOD of CPA for *sea*, *seb*, and *pvl* was 75, 107.5, and 85 ng/μl (Figure 1). Regular PCR was also performed, and expected bands were obtained as well. In a combination of the above results, it was indicated that the analytical sensitivity of CPA is significantly higher than that of conventional PCR.

### Development of CPA Assays

Specificity of the CPA assays was also studied. For the specificity of primers, CPA was performed with any one of the five primers (4s, 5a, 2a/1s, 2a, and 3a) omitted. According to the results, every single primer is strictly and specifically required for the reaction. In addition, the specificity of the set of primers was confirmed by including five MRSA strains with 31 non-MRSA strains. As shown by the results (Table 1), all targets were strictly specific within the species and strains detected. It was indicated that the analytical specificity of CPA is significantly higher than that of conventional PCR.

### Application of the CPA Assays in Artificially Contaminated Food

Limit of detection of CPA was also determined in real food samples, and the LOD was found to be approximately 10^4^ CFU/ml for all three targets (data not shown). Compared with PCR, the sensitivity of CPA, as was demonstrated, is significantly higher than that of PCR. Furthermore, this result was obtained using real food samples, such as Cantonese cake, indicating the applicability of the developed assays.

### Formation of VBNC and Application of PMA-CPA

Formation of VBNC was investigated using the previous procedure. After VBNC formation, the MRSA cells were detected using the PMA-CPA on the expression of *sea*, *seb*, and *pvl*. As PMA was concerned, the concentrate influenced the final results. The concentrate used in this study was 5 μg/ml, as described previously. After the combination of PMA, the samples were first kept in the dark for 15 min and then under the halogen lamp for another 15 min, as described previously. Centrifugation was then performed and DNA was extracted accordingly. PMA-CPA was further performed. In addition, the PMA-CPA was confirmed by using PCR and the VBNC confirmation. According to the results, PMA-CPA showed significant advantages when compared with PCR assays, in terms of rapidity, sensitivity, specificity, and accuracy. Compared with conventional VBNC confirmation methods, the PMA-CPA showed 100% accordance, which demonstrated that the PMA-CPA assays were capable of detecting different toxins in MRSA in VBNC state.

## Discussion

Food safety and foodborne infections and diseases have been a leading hotspot in public health, and foodborne infections or diseases caused by food pathogens have made up the majority of the cases. MRSA has been recently documented to be an important foodborne pathogen, and its recognition as a leading clinical pathogen for some decades. Examples include the carriage of this pathogen in industrial staff and the prevalence of LA-MRSA, which has been clearly pointed out and established to be a foodborne pathogen. Recently, much attention has been paid to the potential role of different retail meat products from regions worldwide. Moreover, several foodborne acquired MRSA outbreaks have also been reported (Voss et al., 2005; Kadariya et al., 2014). Since food products contaminated with MRSA may not exhibit any spoilage appearance or bad smell, it is challenging for consumers to dispose of the contaminated foods prior to consumption. Apart from that, MRSA has exhibited an increasing trend in numerous countries and emerged as an important pathogen accounting for up to 40% of all *S. aureus* isolates in nosocomial infection and caused significant morbidity (Graveland et al., 2011). Capable of producing enterotoxins, it is also commonly found in food poisoning cases. SFP and PVL are both major concerns when it comes to the pathogenesis of MRSA. Consequently, in this study, two major types of SFP, *sea* and *seb*, as well as the *pvl* genes, were selected to be included to develop the related CPA detection assays.

Standard identification for MRSA has been commonly performed in both clinical settings and food routine detection. However, most of such so-called “standards” or “guidelines” or “gold standards” are based on the culturing of MRSA on different medium plates, including selective and non-selective plates. One major problem exists: whether the bacterial cells of MRSA are non-culturable, which, if so, will further lead to ineffective detection on such medium plates. VBNC was first proposed in 1982. A large proportion of the studies have been done on foodborne microorganisms, since once researchers have failed to detect these bacterial cells, the food sample is likely to be mistaken determined to be safe or qualified. In addition, it is very common, not only in foodborne pathogens, but in clinical settings for laboratory researchers to fail to detect or identify the responsible pathogens in apparent infection patients. Culturability-based methodology has been significantly challenged as the “gold standard” when it comes to microbiological identification. Therefore, rapid detection of MRSA is imperative for both MRSA-related foodborne outbreaks and clinical diagnosis. In this study combining the two concerns, a rapid VBNC cells detection method, designated PMA-CPA, was developed and further applied. The PMA-CPA, on the one hand, shows rapidity compared to all culturing methods or even PCR. On the other hand, it shows accuracy when it comes to detection of VBNC cells.

Furthermore, this study is of great importance as the three PMA-CPA methodologies developed in this study are capable of detecting the toxins in the expression of MRSA in the VBNC state, not only within the bacteria themselves—which is distinctive from most currently available studies. The developed PMA-CPA assays were also further confirmed to be applicable with respect to detection of VBNC MRSA cells in real food samples. In addition, CPA has significant advantages when compared with regular PCR, in terms of rapidity, sensitivity, specificity, and simplicity in operation/requirement for equipment. In comparison, food samples are more complicated and contain more potential inhibitory substances than clinical samples. The CPA assays developed in this study, according to our previous experience, are definitely applicable in clinical samples, since they are able to be applied to food samples. In addition, in our experience, for clinical samples, non-culturable isolates are a major concern, as in many cases, conventional culturing methodologies are incapable of identifying the pathogen responsible for infection or diseases. The CPA assays developed in this study are capable of directly detecting those viable cells (even though they are non-culturable), which will significantly aid in the detection of MRSA in clinical samples, especially in the case of non-culturable cells.

## Conclusion

Three CPA assays were developed on three important toxins for MRSA, and in combination with PMA, the PMA-CPA assays were capable of detecting the toxins from the expression of MRSA in the VBNC state. The above assays were further applied in real samples. We have concluded that the PMA-CPA assays developed in this study are capable of detecting MRSA toxins in the VBNC state, representing success for the first time in the detection of toxins in the VBNC state.

## Data Availability Statement

The raw data supporting the conclusions of this article will be made available by the authors, without undue reservation.

## Author Contributions

JL and KW conceived of the study and participated in its design and coordination. HJ, LC, MY, QY, XL, and YY performed the experimental work and collected the data. LZ and TH analyzed the data and wrote the manuscript. All authors read and approved the final manuscript.

## Conflict of Interest

The authors declare that the research was conducted in the absence of any commercial or financial relationships that could be construed as a potential conflict of interest.
